# Assessing *Venturia inaequalis* Response to Common Fungicides in Morocco

**DOI:** 10.3390/jof11070493

**Published:** 2025-06-29

**Authors:** Safae Gouit, Safae Chiadmi, Khadija Goura, Ikram Legrifi, Moussa El Jarroudi, Zineb Belabess, Abdessalem Tahiri, Abderrahim Lazraq, Mohammed Baala, Rachid Lahlali

**Affiliations:** 1 Phytopathology Unit, Department of Plant Protection, École Nationale d’Agriculture de Meknès, Km10, Rte Haj Kaddour, BP S/40, Meknes 50001, Morocco; safae.gouit@gmail.com (S.G.); schiadmi@enameknes.ac.ma (S.C.); k.goura@edu.umi.ac.ma (K.G.); ikramlegr@gmail.com (I.L.); atahiri@enameknes.ac.ma (A.T.); mbaala@enameknes.ac.ma (M.B.); 2 Laboratory of Functional Ecology and Environmental Engineering, Sidi Mohamed Ben Abdellah University, Route d’Imouzzer, P.O. Box 2202, Fez 30000, Morocco; lazraqab@gmail.com; 3 Department of Sciences and Environmental Management, University of Liege, 6700 Arlon, Belgium; 4 Plant Protection Laboratory, Regional Center of Agricultural Research of Meknes, National Institute of Agricultural Research, km 13, Route Haj Kaddour, Meknes 50001, Morocco; zineb.belabess@inra.ma

**Keywords:** apple scab, sensitivity, thiophanate-methyl, trifloxystrobin, difenoconazole, disease management, fungicide resistance

## Abstract

Apple scab, caused by *Venturia inaequalis*, remains a major challenge for apple production in Morocco, where disease management heavily depends on fungicide applications. However, increasing reports of resistance have raised concerns about the long-term efficacy of commonly used products and the economic sustainability of apple orchards. In this study, we evaluated the sensitivity of five *V. inaequalis* isolates from the Fes-Meknes region, a key apple-producing area in Morocco, to three fungicides: difenoconazole, trifloxystrobin, and thiophanate-methyl. The identity of the isolates was confirmed based on both morphological characteristics and by molecular analysis of the ITS region. In vitro and in vivo assays revealed significant differences in isolate responses. Difenoconazole consistently showed the highest efficacy, with EC_50_ values ranging from 0.05 to 1.46 µg/mL, and preventive applications reducing disease severity by up to 85.8% at 10 µg/mL. In contrast, trifloxystrobin and thiophanate-methyl exhibited much higher EC_50_ values (2.94–29.62 µg/mL and 14.84–1237.20 µg/mL, respectively), indicating widespread resistance, particularly to thiophanate-methyl, whose curative and preventive efficacy rarely exceeded 44%. Preventive treatments were significantly more effective than curative applications for all fungicides tested. These findings highlight the urgent need to revise apple scab management strategies in Morocco, including the rotation of fungicides with different modes of action and the integration of non-chemical approaches. Broader sensitivity monitoring and the use of molecular diagnostics are recommended to better inform sustainable disease control programs.

## 1. Introduction

Apple scab, caused by *Venturia inaequalis* (Cooke) G. Winter, is one of the most devastating diseases affecting cultivated apples (*Malus × domestica* Borkh.) worldwide. It affects leaves, flowers, buds, shoots, and fruits, leading to substantial economic losses. In the absence of effective control measures, production losses can reach up to 70% of market value and may impact up to 100% of the yield [1], particularly under weather conditions favorable to disease development [2,3,4,5]. *V. inaequalis* is prevalent in nearly all regions where commercial apples are cultivated. However, the disease is especially severe in temperate regions with cool, moist climates, particularly during early spring [5].

Despite various cultural practices and the limited availability of resistant cultivars, chemical control remains the primary method for managing apple scab. Fungicide sprays are typically applied from bud break at regular intervals of 7 to 10 days, continuing until the risk of infection subsides. Protectant fungicides are used early in the season, when foliage is sparse or when infections can be predicted. Curative fungicides are applied when protective treatments fail to prevent infection [6].

A variety of fungicide classes are currently in use, either individually or in combination, with growers often applying between 15 and 25 treatments during the growing season [3,4]. The three most widely used classes of systemic fungicides are methyl benzimidazole carbamates (MBCs), demethylation inhibitors (DMIs), and quinone outside inhibitors (QoIs) [7]. Each class has a distinct mode of action (MoA). MBC fungicides, such as thiophanate-methyl, inhibit mitosis and fungal cell division by binding to the *β-tubulin* gene [8]. DMI fungicides, such as difenoconazole, disrupt sterol biosynthesis in fungal membranes by targeting the *Cyp51* gene [9]. QoI fungicides, such as trifloxystrobin, block mitochondrial respiration by binding to the quinone-oxidizing site (QoI site) of the cytochrome bc1 (*cyt b*) enzyme complex [10].

Therefore, the risk of fungicide resistance represents a major challenge in managing apple scab, as *V. inaequalis* is known for its high potential to develop resistance [11]. In most cases, competitive selection has led to the emergence of fungicide-resistant variants, posing a serious threat to effective disease control [12]. For instance, dodine was introduced in 1959 for scab management, but documented cases of resistance appeared within a decade [13]. Numerous studies have reported resistance in *V. inaequalis* to various fungicides, including difenoconazole, thiophanate-methyl, dodine, benomyl, myclobutanil, azoxystrobin, trifloxystrobin, and kresoxim-methyl [14,15,16,17,18,19,20,21].

In Morocco, apple orchards cover approximately 51,800 hectares, accounting for nearly 20% of the country’s area dedicated to rosaceous fruit trees [22]. In major production regions such as Fes-Meknes, apple scab remains the primary phytosanitary challenge for growers. To combat this persistent threat, Moroccan farmers often apply 12 to 20 fungicide treatments per season, resulting in substantial costs and increasing the risk of resistance development [23,24]. Currently, more than 18 approved active ingredients are available in the country for managing this disease [25]. Farmers are encouraged to reduce the frequency of fungicide applications and rotate products with different modes of action to limit the development of resistance [1].

Despite these efforts, Moroccan apple growers often report dissatisfaction with the effectiveness of fungicide treatments against apple scab. Concerns about reduced sensitivity are frequently raised, highlighting a critical gap in understanding the development of resistance in *V. inaequalis* populations in Morocco [7]. Moreover, assessing sensitivity parameters is essential for preventing resistance development to commonly used fungicide classes. Therefore, the primary objective of this study was to evaluate the sensitivity of *V. inaequalis* isolates from major apple-producing provinces in the Fes Meknes region of Morocco to thiophanate-methyl, difenoconazole, and trifloxystrobin. In addition, we assessed the severity of apple scab on detached leaves under controlled in vivo conditions.

## 2. Materials and Methods

### 2.1. Study Area and Sampling Strategy

**Figure 1:**
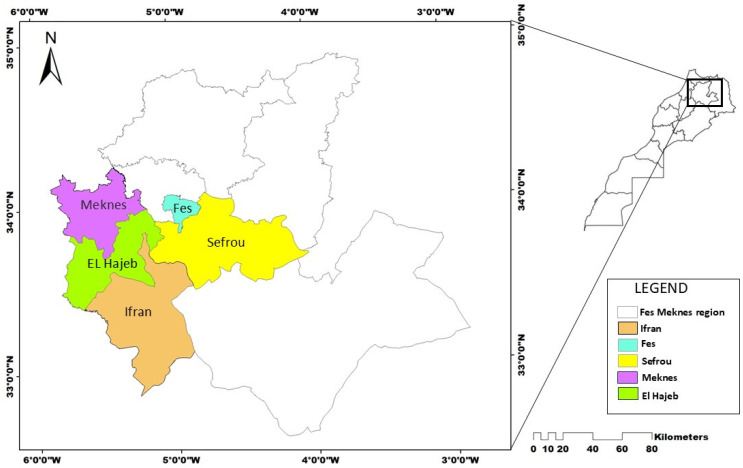
Map showing the study area: Meknes, Ifrane (Azrou), Sefrou (Imouzzer), and El Hajeb, the main apple-producing provinces in the Fes-Meknes region.

This study was conducted in three major apple-producing provinces within the Fes-Meknes region of Morocco (Figure 1), which together constitute the country’s main apple-growing areas. These provinces were selected based on agronomic and phytosanitary criteria provided by local growers and agricultural technicians, including the frequency of fungicide applications, persistence of scab symptoms, and diversity of orchard management practices.

Sampling was carried out during the peak scab season using the simple random sampling method described by Bora et al. [26]. Both commercial and experimental apple orchards were visited, and plant organs (leaves and/or fruits) showing typical symptoms of apple scab (caused by *V. inaequalis*) were collected. Each sampling site was georeferenced using a GPS device, and a unique code was assigned to each sample based on its province and district of origin (Table 1).

In each orchard, symptomatic leaves and fruits were carefully collected, with a minimum of 10 leaves or fruits per tree and at least three trees sampled per site to ensure representativeness. Selection was based on visible scab lesions, and care was taken to avoid sampling from trees or zones recently treated with fungicides, as verified through orchard management records. For the untreated control, samples were specifically collected from an experimental plot at the National School of Agriculture of Meknes, which has not received fungicide treatments for at least five years.

All collected samples were individually packed in sterile bags and transported in a cooled container to the laboratory. They were stored at 4 °C and processed within 24 h to preserve pathogen viability.

### 2.2. Pathogen Isolation

Isolation of *V. inaequalis* was performed following a rigorous and reproducible protocol as described by Kavak et al. [27]. Small sections of infected tissue, including both symptomatic areas and adjacent healthy-looking zones, were excised under sterile conditions. Each fragment was placed in 10 mL of sterile distilled water and gently shaken at 120 rpm for 30 min to release conidia. The resulting suspension was filtered through sterile gauze to remove debris, and 100 µL of the filtrate was spread onto 2% water agar (Bacto Agar, Difco) supplemented with streptomycin and gentamicin, each at a concentration of 25 mg/L, to inhibit bacterial growth.

Plates were incubated in the dark at 20 ± 2 °C for up to two weeks. Germinating conidia were identified under a light microscope and individually transferred to potato dextrose agar (PDA) plates containing the same antibiotics [12]. These subcultures were incubated under the same conditions and regularly monitored for purity. Only isolates displaying the typical morphological characteristics of *V. inaequalis* were retained for further analysis.

To ensure the reliability of downstream assays, all isolates were subcultured at least twice. Pure cultures were preserved as mother stocks on slants at 4 °C and also cryopreserved in 20% glycerol at −80 °C.

In total, approximately 30 presumptive *V. inaequalis* isolates were obtained. To confirm pathogenicity, a subset of isolates was tested on detached apple leaves, following the protocols of Nicholson et al. [28] and Olivier and Lespinasse [29]. After four weeks of incubation in a humid chamber at 18 °C, isolates were classified based on their ability to induce characteristic scab lesions and sporulation. The five most virulent isolates, representing distinct geographic origins and orchard management practices (four from commercial orchards and one from an untreated experimental orchard) [22], were selected for further study.

### 2.3. Identification of the Fungal Species: Morphological Observations and Molecular Analysis

The identification of *V. inaequalis* isolates was based on a dual approach combining morphological assessment and molecular techniques to ensure both precision and reliability. Each isolate was first evaluated macroscopically by observing colony characteristics on PDA medium, focusing on color, texture, and growth patterns after incubation at 20 ± 2 °C in darkness for several weeks. In addition, microscopic analysis was performed using a light microscope to examine key diagnostic structures, including conidia and ascospores, which are essential for species confirmation

For molecular confirmation, genomic DNA was extracted from seven-day-old mycelium using a modified CTAB protocol [30], and DNA quality was assessed by spectrophotometry (A260/A280 ratio ≥ 1.8) and verified by agarose gel electrophoresis. The internal transcribed spacer (ITS) region of the ribosomal DNA was amplified by PCR using the universal primers ITS1 (5′-TCCGTAGGTGAACCTGCGG-3′) and ITS4 (5′-TCCTCCGCTTATTGATATGC-3′), following the protocol described by White et al. [31]. Each 25 µL PCR reaction contained 2.5 µL of 10× buffer, 0.5 µL of dNTPs (10 mM), 1 µL of each primer (10 µM), 0.2 µL Taq DNA polymerase (5 U/µL), 2.5 µL of genomic DNA, and nuclease-free water to adjust the final volume. The thermal cycling conditions included an initial denaturation at 95 °C for 10 min, followed by 35 cycles of 95 °C for 35 s, 55 °C for 1 min, and 72 °C for 2 min, with a final extension at 72 °C for 10 min.

PCR products were visualized on 1.5% agarose gels stained with ethidium bromide. Amplicons of the expected size were purified using a commercial kit and sequenced using the Sanger method. The resulting sequences were edited and aligned using BioEdit software, then compared to reference sequences in the GenBank database using the BLASTn algorithm. Only sequences showing ≥97% identity with *V. inaequalis* were retained. Representative sequences from this study were deposited in GenBank (PQ661209 and PQ661203).

### 2.4. In Vitro Fungicide Sensitivity Tests

To evaluate the sensitivity of *V. inaequalis* isolates to three commercial fungicides—Tresor 250 EC (difenoconazole, 250 g/L), Flint 50 WG (trifloxystrobin, 50%), and Pelt 70 WG (thiophanate-methyl, 70%)—an experimental study was conducted under controlled conditions [32]. Each fungicide was dissolved in sterile distilled water according to the manufacturer’s instructions to ensure complete solubilisation and uniform dispersion. The presence of surfactants and dispersants in the EC and WG formulations facilitated homogeneous mixing with molten, cooled PDA at approximately 45 °C. Five concentrations of each active ingredient were tested: 0.05, 0.5, 1, 5, and 10 µg/mL. After solidification, all media were visually inspected to confirm the absence of precipitation or formulation incompatibility.

Mycelial plugs (5 mm in diameter) were excised from the actively growing margins of 14-day-old colonies and placed upside down at the center of either unamended PDA (control) or PDA amended with one of the fungicides. For each isolate and concentration, four replicate plates were prepared, and the entire experiment was independently repeated to confirm the reproducibility. Plates were incubated in darkness at 20 ± 2 °C for up to 28 days. To minimize positional effects, plates were regularly rotated within the incubator.

Colony diameters were measured in two perpendicular directions using a digital caliper (accuracy ± 0.01 mm). The percentage of mycelial growth inhibition in the presence of each fungicide was calculated relative to the untreated control. The effective concentration required to inhibit 50% of mycelial growth (EC_50_) was then estimated graphically for each isolate and fungicide.

The resistance status of the isolates was determined based on international EC_50_ thresholds defined by the Fungicide Resistance Action Committee (FRAC) guidelines, with specific criteria for each fungicide: difenoconazole, resistant if EC_50_ > 0.1 µg/mL; trifloxystrobin, resistant if EC_50_ > 2 µg/mL; thiophanate-methyl, moderately resistant between 10 and 100 µg/mL and highly resistant if EC_50_ > 100 µg/mL.

### 2.5. In Vivo Test of Fungicide Efficacy Against V. inaequalis on Detached Leaves

This test was conducted on young detached leaves of the Golden Delicious apple variety, which showed no visible disease symptoms. Leaves were collected from a dedicated experimental plot managed without any phytosanitary treatments to ensure the absence of fungicide residues.

The pathogenicity protocol described by Nicholson et al. [28], used to assess the severity of *V. inaequalis* on detached leaves, was followed precisely. This protocol included surface inoculation, preventive and curative fungicide treatments, and subsequent lesion evaluation according to fungicide concentration.

In both preventive and curative treatments, detached leaves were immediately immersed in water, gently cleaned to remove leaf hairs and debris, and rinsed under running tap water for 15 min. Following this, leaves were disinfected by immersion in sterile distilled water for several minutes to minimize contamination risk before treatment and inoculation. The petiole was trimmed close to the leaf base to enhance optimal vascular contact with water in a petri dish. After three rinses with sterile water, each leaf was placed adaxial side up, ensuring that the cut petiole was in contact with 1% water agar in the petri dish. No yellowing of the detached leaves was observed during or after the immersion and washing steps, as processing was immediate and immersion time was kept short to preserve leaf vitality.

For the preventive treatment, each leaf was immersed in a distilled water solution (DSW) containing the tested fungicide concentration for approximately two minutes. This immersion time was standardized based on phytotoxicity assays, which showed no visible leaf damage at this duration. Commercial formulations were directly diluted in distilled water according to the manufacturer’s recommendations and gently agitated to ensure maximum dispersion. Although some active ingredients (e.g., trifloxystrobin) have limited intrinsic solubility in water, the presence of surfactants and dispersants in commercial formulations ensured adequate mixing and coverage under experimental conditions. Immersion was performed over spraying to guarantee uniform and complete coverage of the leaf surface, as recommended in laboratory-based detached leaf assay protocols. Following treatments, the leaves were placed adaxial side up on a water-gelled medium in petri dishes and allowed to air-dry for approximately two hours before inoculation. Inoculation was carried out using a *V. inaequalis* spore suspension adjusted to a concentration of 1 × 10^6^ spores/mL. Spore concentration was determined using a Bürker counting chamber, and the final suspension was diluted with sterile distilled water to reach the target concentration.

For the curative treatment, disinfected leaves were first placed in petri dishes under sterile conditions, with the upper surface kept dry under a laminar flow hood. The spore suspension was applied to the surface and allowed to incubate for two hours. After this period, a specific fungicide concentration was applied to each leaf using a handheld atomizer calibrated to deliver a fine, uniform mist, ensuring consistent coverage across all treated leaves.

In both treatments, 50 μL of the spore suspension (1 × 10^6^ spores/mL) was applied to the upper surface of each leaf in fine droplets, ensuring minimal runoff [33]. Each combination of *V. inaequalis* isolate and fungicide concentration was tested on four leaves (four replicates), with two leaves per petri dish. Control leaves were treated with an equal volume of DSW instead of fungicide.

Petri dishes were sealed with Parafilm and placed in a growth chamber set at 18 ± 2 °C for a 16 h photoperiod provided by white fluorescent lamps [34]. The fluorescent lamps used for incubation delivered a light intensity of 120 µmol/m^2^/s and a spectrum covering 400–700 nm, ensuring optimal conditions for fungal development and reproducibility of the assay. The experiment was repeated twice.

To evaluate the sensitivity of *V. inaequalis*, inoculated leaves were assessed four weeks after inoculation, when control leaves were nearly fully infected. Disease severity was visually estimated as the percentage of leaf surface affected by the fungus, using a rating scale developed by Calenge et al. [35]. The scale consists of seven categories: (0) no visible lesions; (1) <1% of the leaf surface affected; (2) 1–5%; (3) 5–10%; (4) 10–25%; (5) 25–50%; (6) 50–75%; and (7) 75–100%.

Fungicide efficacy was calculated using the formula proposed by Tehon et al. [36]:

Fungicide Efficacy = ((A − B)/A) × 100(1)

where A is the disease severity in the control leaves, and B is the severity in the treated leaves.

### 2.6. Data Analysis

The effective concentration required to inhibit 50% inhibition of mycelial growth (EC_50_) for each fungicide and isolate was determined using XLSTAT software (version 2018, Addinsoft, Paris, France). A log-logistic regression model was fitted to arcsine square root-transformed inhibition data against log_10_-transformed fungicide concentrations following the Fungicide Resistance Action Committee (FRAC) Guidelines [37]. For both in vitro and in vivo experiments, data were subjected to analysis of variance (ANOVA) using SPSS software (version 26.0, IBM Corp., Armonk, NY, USA) to evaluate the effects of fungicide, concentration, and isolate on disease severity and mycelial growth inhibition.

Where significant effects were detected, means were separated using multiple comparison tests, including Student–Newman–Keuls (S-N-K), Dunnett’s, and Duncan’s multiple range tests at a significance level of *p* ≤ 0.05, as commonly applied in fungicide efficacy studies. The normality of residuals and homogeneity of variances were verified before performing ANOVA to ensure that statistical assumptions were met. All statistical analyses were performed separately for the in vitro and in vivo datasets.

## 3. Results

### 3.1. Isolate Identification

**Figure 2:**
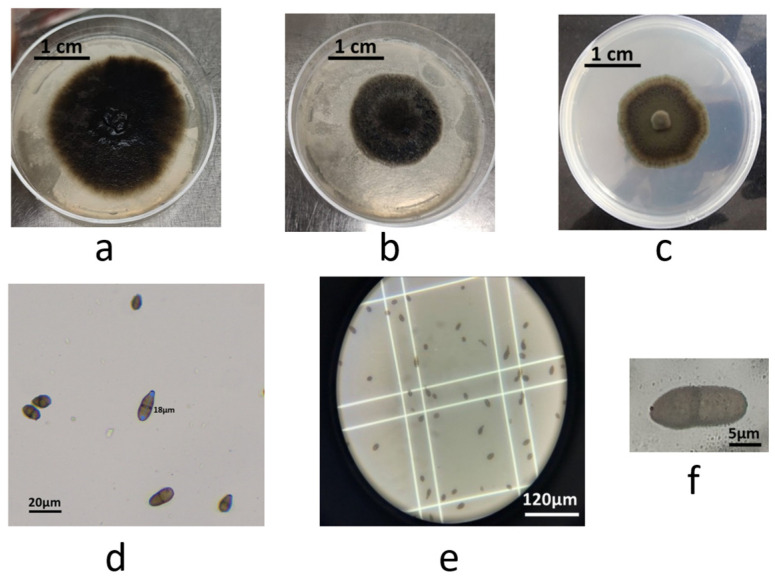
Morphological and microscopic characteristics of representative *V. inaequalis* isolates from Moroccan apple orchards. (**a**) Colony morphology of isolate ViIF01 on PDA after 28 days of incubation at 20 ± 2 °C in darkness (surface view). (**b**) Colony morphology of isolate ViAZ on PDA after 14 days at 20 ± 2 °C in darkness (surface view). (**c**) Colony morphology of isolate ViIM on PDA after 14 days at 20 ± 2 °C in darkness (surface view). (**d**) Unicellular, ellipsoid conidia of isolate ViEN01 (scale bar = 10 µm, ×400). (**e**) Unicellular, ellipsoid conidia of isolate ViEN01 (scale bar = 10 µm, ×1000). (**f**) Bicellular ascospore of isolate ViHA (scale bar = 10 µm, ×1000).

All five *V. inaequalis* isolates exhibited highly similar morphological characteristics on PDA medium. Colonies grew slowly, initially appearing cottony white and became more compact with an olive-to-black coloration as they aged (Figure 2a–c). These features are consistent with previously described colony morphologies of *V. inaequalis* [38,39]. Microscopic examination revealed predominantly unicellular, ellipsoid conidia measuring 12–19 µm in length with occasional septation (Figure 2d,e), as well as the presence of bicellular ascospores (Figure 2f), supporting accurate identification at the species level [1,39].

To confirm species identity, the ITS region of ribosomal DNA from two representative isolates (ViHA and ViAZ) was sequenced. BLAST v 2.15.0 analysis revealed ≥97% identity with reference *V. inaequalis* sequences (e.g., MW810284; KY 556629) [1]. The sequences were deposited in GenBank under accession numbers PQ661203 (ViHA) and PQ661209 (ViAZ). No significant morphological or molecular differences were observed between isolates from commercial orchards and the untreated orchard, indicating high genetic and phenotypic similarity across the sampled populations.

These findings confirm that all five isolates belong to *V. inaequalis*, based on both morphological and molecular evidence.

### 3.2. In Vitro Evaluation of Fungicide Effectiveness and EC_50_ Determination

**Figure 3:**
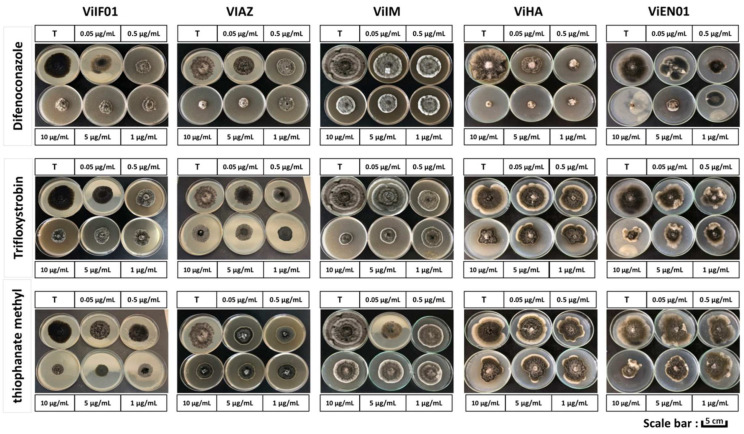
In vitro effect of three fungicides at five concentrations (0.05, 0.5, 1.0, 5.0, 10.0 µg/mL) on the mycelial growth of five *V. inaequalis* isolates after 28 days of incubation on PDA at 20 ± 2 °C. For each isolate (ViIF01, ViAZ, ViIM, ViHA, ViEN01), colony morphology is shown across increasing fungicide concentrations. A progressive reduction in colony diameter with higher fungicide concentration illustrates the dose-dependent inhibition observed for all fungicides. Difenoconazole exhibited the highest inhibitory effect, whereas trifloxystrobin and thiophanate-methyl showed reduced efficacy, particularly against ViAZ and ViIM. All images were taken after 28 days of incubation. T: untreated control. Fungicide concentrations are expressed in µg/mL. Scale bar = 5 cm (petri dish diameter = 90 mm). Quantitative inhibition data are shown in Table 2; EC_50_ values for each isolate and fungicide are presented in Table 3.

After 28 days of incubation on PDA medium at 20 ± 2 °C, the five *V. inaequalis* isolates exhibited marked differences in their sensitivity to difenoconazole, trifloxystrobin, and thiophanate-methyl. Figure 3 provides a visual overview of colony morphology and growth inhibition for each isolate and fungicide combination across all tested concentrations, while Table 2 presents the corresponding quantitative inhibition data (mean ± SD, *n* = 4) along with statistical groupings.

Difenoconazole exhibited the highest overall efficacy. Inhibition rates ranged from 20.2% ± 2.8% (ViAZ at 0.05 µg/mL) to 94.6% ± 1.7% (ViEN01 at 10 µg/mL), with the ViHA isolate showing particularly high sensitivity (inhibition increasing from 50.3% ± 1.7% to 91.5% ± 3.2%). In contrast, ViIF01 and ViIM were less sensitive, with maximum inhibition values of 71.4% ± 2.4% and 74.4% ± 1.6% at 10 µg/mL, respectively.

Trifloxystrobin was less effective, with inhibition not exceeding 62.8% ± 2.3% (ViIF01 at 10 µg/mL), and most isolates exhibited moderate to low sensitivity. For instance, ViAZ and ViIM showed inhibition rates below 55% even at the highest concentration.

Thiophanate-methyl was the least effective overall, with inhibition rarely exceeding 55% at 10 µg/mL. The ViAZ isolate was particularly insensitive, showing only 25.7% ± 2.0% inhibition at the highest dose. In comparison, ViIM reached 35.1% ± 1.9% and ViIF01 achieved 54.3% ± 1.9% at the same concentration. These differences are clearly visible in Figure 3, where colony size reduction is most pronounced with difenoconazole and minimal with thiophanate-methyl, particularly for ViAZ and ViIM.

To quantify these observations, EC_50_ values were calculated for each isolate and fungicide (see Table 3). Difenoconazole EC_50_ values ranged from 0.05 ± 0.01 µg/mL (ViHA, group a, sensitive) to 1.46 ± 0.15 µg/mL (ViIM, group d, resistant). For trifloxystrobin, EC_50_ values ranged from 2.94 ± 0.29 µg/mL (ViIF01, group a, resistant) to 29.62 ± 2.96 µg/mL (ViIM, group e, resistant), and for thiophanate-methyl, values ranged from 14.84 ± 1.48 µg/mL (ViIF01, group a, moderately resistant) to 1237.20 ± 12.37 µg/mL (ViAZ, group e, highly resistant).

Table 3 summarizes these EC_50_ values, their statistical groupings, and resistance classification according to international thresholds. Notably, only ViHA was classified as sensitive to difenoconazole (EC_50_ < 0.1 µg/mL), while all isolates were resistant to trifloxystrobin (EC_50_ > 2 µg/mL) and at least moderately resistant to thiophanate-methyl (EC_50_ > 10 µg/mL).

Statistical analysis revealed significant differences among fungicides, concentrations, and isolates (ANOVA, *p* ≤ 0.05; Duncan’s test). These findings highlight the widespread occurrence of multi-fungicide resistance in Moroccan *V. inaequalis* populations and emphasize the urgent need for integrated disease management strategies.

### 3.3. In Vivo Evaluation of Fungicide Efficacy on Detached Apple Leaves

The in vivo sensitivity of five *V. inaequalis* isolates to difenoconazole, trifloxystrobin, and thiophanate-methyl was assessed on detached apple leaves after 4 weeks of incubation. Disease severity and fungicide efficacy varied significantly among isolates, concentrations, and application methods (preventive vs. curative). Table 4 summarizes disease severity and calculated efficacy for each treatment combination.

Control disease severity remained consistently high across all isolates, ranging from 98.81% ± 0.5% (ViIF01) to 99.75% ± 0.5% (ViAZ and ViEN01), confirming successful pathogen establishment and leaf susceptibility under experimental conditions.

Difenoconazole demonstrated the highest efficacy in both preventive and curative applications. In preventive treatments, disease severity was significantly reduced, reaching as low as 14.2% ± 0.9% (ViEN01 at 10 µg/mL), corresponding to 85.5% ± 0.9% efficacy. The ViEN01 isolate was the most sensitive, with efficacy increasing from 41.5% ± 2.1% at 0.05 µg/mL to 85.5% ± 0.9% at 10 µg/mL. Curative applications were less effective, but still significant, with a maximum efficacy of 72.3% ± 1.4% (ViEN01 at 10 µg/mL). In contrast, ViIF01 showed lower sensitivity, with preventive efficacy reaching only 52.5% ± 0.9% at 10 µg/mL. Statistical analysis revealed significant differences among concentrations (Duncan’s test, *p* ≤ 0.05), with treatments at 5 and 10 µg/mL forming distinct groups (groups a and b) compared to lower concentrations

Trifloxystrobin showed moderate efficacy, with notable differences between preventive and curative applications. Preventive treatments achieved a maximum efficacy of 54.5% ± 0.9% (ViIF01 at 10 µg/mL), whereas curative treatments were markedly less effective, not exceeding 42.6% ± 1.4% under the same conditions. ViHA exhibited reduced sensitivity, with maximum preventive efficacy of only 49.4% ± 2.5% at 10 µg/mL, while ViAZ was even less responsive, with only 31.1% ± 0.9% at the same concentration.

Thiophanate-methyl exhibited the lowest efficacy across all isolates and concentrations. In preventive applications, maximum efficacy reached 43.9% ± 0.9% (ViIF01 at 10 µg/mL), while curative treatments did not exceed 36.5% ± 1.4% (ViIF01 at 10 µg/mL). ViAZ was particularly insensitive, with disease severity remaining above 77% even at the highest concentration, resulting in only 22.5% ± 0.9% preventive and 19.9% ± 1.0% curative efficacy at 10 µg/mL. In comparison ViEN01 achieved 41.5% ± 2.0% efficacy with preventive application at the same dose.

Preventive applications were significantly more effective than curative ones across all fungicides and isolates (ANOVA, *p* < 0.001). This effect was most pronounced with difenoconazole, where preventive efficacy exceeded curative efficacy by 10–13 percentage points at 10 µg/mL, followed by trifloxystrobin (5–12 points) and thiophanate-methyl (2–10 points). The superior performance of preventive applications highlights the importance of protective fungicide use in apple scab management.

Clear dose–response relationships were observed for all fungicides, with efficacy increasing significantly with concentration. Duncan’s multiple range test identified distinct statistical groupings, particularly at higher concentrations (5 and 10 µg/mL), where maximum efficacy was reached.

These in vivo findings largely corroborate the in vitro results, with difenoconazole consistently showing the highest efficacy and thiophanate-methyl the lowest. However, absolute efficacy values differed among assays, reflecting the greater complexity of plant–pathogen–fungicide interactions under realistic host-based conditions compared to culture-based systems.

## 4. Discussion

This study provides a comprehensive assessment of the sensitivity of *V. inaequalis* isolates from the Fes-Meknes region to three major classes of fungicides, offering new insights into the current status of apple scab management in Morocco. Our results show that difenoconazole remains the most effective fungicide among those tested, both in vitro and in vivo, with EC_50_ values ranging from 0.05 to 1.46 µg/mL, often below or close to the practical resistance threshold established by FRAC. However, the finding that certain isolates, such as ViIM, exhibit EC_50_ values exceeding 1 µg/mL is a clear indication of emerging reduced sensitivity, an alarming trend that has also been reported in other apple-growing regions worldwide [7,40].

Recent studies in the northwestern Himalayas of India have similarly documented a shift in sensitivity to difenoconazole, with EC_50_ values ranging from 0.001 to 0.584 µg/mL, and have identified the Y133F mutation in the *CYP51A1* gene as a contributing factor to reduced susceptibility in resistant and shifted isolates [41,42]. In Serbia, for example, EC_50_ values for difenoconazole ranged from 0.081 to 0.362 µg/mL in resistant isolates, with resistance factors up to 22.6 compared to sensitive populations, highlighting the global challenge of DMI resistance [43]. Compared to these studies, our EC_50_ values (0.05 to 1.46 µg/mL) suggest a potentially higher level of reduced sensitivity in Moroccan isolates, particularly in the case of ViIM. Furthermore, overexpression of the *CYP51A1* gene has been shown to significantly contribute to resistance to difenoconazole and other DMI fungicides, with resistant isolates exhibiting up to 13-fold increases in gene expression compared to sensitive ones [44]. This finding underscores the importance of continued monitoring and the judicious use of DMI fungicides to prevent further erosion of their efficacy.

The study also reveals a marked difference in efficacy between preventive and curative applications across all fungicides, with preventive treatments consistently achieving higher levels of disease control. For example, difenoconazole reduced disease severity by up to 85.5% on detached leaves at 10 µg/mL when applied preventively compared to 72.3% in curative mode for the most sensitive isolate, ViEN01. This observation aligns with international recommendations emphasizing the importance of timely, preventive interventions to maximize fungicide performance and delay resistance development [36,44]. These results highlight the need for Moroccan growers to optimize spray timing and take orchard-specific risk factors into account when designing disease management programs.

Trifloxystrobin, which was previously reported as highly effective in the region [7], now shows widespread resistance among the tested isolates, with all EC_50_ values exceeding 2 µg/mL. This rapid decline in sensitivity is consistent with findings from other Mediterranean countries, where the G143A mutation in the *cyt b* gene has been identified as a major driver of QoI resistance [45]. The G143A mutation, a single-nucleotide polymorphism in the *cytochrome b* gene resulting in the substitution of glycine with alanine at position 143, is widely recognized as the primary mechanism of QoI resistance in *V. inaequalis* and can be efficiently detected using pyrosequencing methods [46].

The current findings strongly suggest that the continued use of trifloxystrobin as a stand-alone product is no longer advisable in Morocco. Instead, its use should be limited and always combined with multisite fungicides to help slow the spread of resistance.

Thiophanate-methyl exhibited the lowest efficacy of the three fungicides tested, with several isolates classified as highly resistant based on EC_50_ values exceeding 100 µg/mL. This observation aligns with its recent withdrawal from the Moroccan market and previous reports of widespread MBC resistance in the region [7]. The high levels of resistance likely result from historical overuse since its introduction in the early 1970s, with resistance documented as early as 1974 in Australia and 1976 in Michigan, reflecting a global pattern of rapid resistance development to benzimidazoles shortly after their widespread adoption [47]. Molecular studies have identified point mutations at codons 198 and 200 of the *β-tubulin* gene as the primary mechanisms of resistance to thiophanate-methyl and other MBC fungicides. Substitutions such as E198A (glutamic acid to alanine) and F200Y (phenylalanine to tyrosine) confer high levels of resistance in various fungal pathogens, including *V. inaequalis* [48]. The persistence of such high resistance levels, even after the product’s withdrawal in Morocco, underscores the long-term impact of selection pressure and highlights the need for careful stewardship of any future single-site fungicides.

The diversity of orchard management histories represented by the five isolates in this study, including both commercial and untreated orchards, provides a meaningful cross section of the current *V. inaequalis* population. Although the sample size is limited, this approach is consistent with recent Moroccan and international studies aimed at establishing baseline sensitivity profiles under practical constraints [7,40]. The findings underscore the value of combining both laboratory and in vivo assays to better capture the complexity of fungicide-pathogen interactions.

Looking ahead, expanding resistance monitoring efforts to include a larger and more diverse set of isolates from different regions and management systems will be essential for refining recommendations and anticipating emerging resistance trends. The integration of molecular diagnostics, such as routine screening for mutations in the *CYP51*, *cyt b*, and *β-tubulin* gens, will further enhance the capacity to track resistance evolution and tailor fungicide strategies accordingly [40]. Moreover, promoting integrated disease management approaches—including reducing the total number of fungicide applications, rotating modes of action, and incorporating non-chemical measures such as resistant cultivars or biological control agents—will be critical for maintaining effective control of apple scab control in Morocco [22,37].

In summary, this study underscores the urgent need for adaptive, evidence-based management of apple scab in Morocco. While difenoconazole remains effective against most isolates, the emergence of reduced sensitivity highlights the importance of continuous monitoring and diversified control strategies. By integrating local data with international best practices, Moroccan apple growers can better protect the long-term health and productivity of their orchards.

## 5. Conclusions

This study provides the first comprehensive assessment of the sensitivity of *V. inaequalis* isolates from Moroccan orchards to three major classes of fungicides. The results show that difenoconazole remains the most effective option for controlling apple scab both in vitro and in vivo, although early signs of reduced sensitivity in certain isolates underscore the need for enhanced monitoring. In contrast, widespread resistance to trifloxystrobin and thiophanate-methyl highlights the limitations of single-site fungicides and calls for a reassessment of current management strategies. Although the study is limited by the small number of isolates tested, it offers a solid foundation for future resistance monitoring. Expanding surveys to include more orchards and integrating molecular diagnostic tools will be critical for developing sustainable disease control programs. Moreover, in light of the challenges posed by climate change, which extends the periods of risk for scab infection, the use of decision support tools, such as predictive models and weather-based forecasts, is strongly recommended to optimize treatment timing. Finally, the adoption of diversified approaches, including resistant cultivars, improved cultural practices, and biocontrol agents, is essential to reduce reliance on fungicides and to ensure the long-term sustainability of apple production in Morocco.

## Figures and Tables

**Table 1:** Characteristics of *Venturia inaequalis* isolates used in this study, including isolate codes, sampling locations, GPS coordinates, and orchard production systems.

Isolate Code	Province	GPS Coordinates of the Orchard	Apple Production Systems
ViIF01	Ifrane	33°36′35″ N 5°9′45″ W	Commercial orchard
ViAZ	Azrou	33°22′11″ N 5°23′25″ W	Commercial orchard
ViIM	Immouzer	33°46′4″ N 5°0′58″ W	Commercial orchard
ViHA	EL Hajeb	33°49′49″ N 5°9′14″ W	Commercial orchard
ViEN01	Meknes	33°50′34.70″ N, 5°28′35.22″ W	An untreated experimental orchard

**Table 2:** Percentage of mycelial growth inhibition (means ± standard deviation) of five *V. inaequalis* isolates exposed to three fungicides at different concentrations, after 28 days of incubation on PDA at 20 ± 2 °C.

Fungicide	Concentration (µg/mL)	ViIF01	ViAZ	ViIM	ViHA	ViEN01
Difenoconazole	0.05	38.96 ± 2.60 b	20.19 ± 2.79 c	20.25 ± 3.08 c	50.33 ± 1.67 a	24.32 ± 3.46 c
	0.5	49.80 ± 2.93 b	45.67 ± 2.38 b	35.70 ± 2.56 c	67.56 ± 1.54 a	54.05 ± 3.10 b
	1.0	53.14 ± 2.71 b	56.46 ± 3.28 b	40.25 ± 2.64 c	74.75 ± 3.17 a	64.19 ± 2.42 b
	5.0	62.51 ± 2.59 b	80.40 ± 3.43 a	62.66 ± 3.35 b	81.44 ± 3.06 a	77.03 ± 3.06 a
	10.0	71.45 ± 2.35 b	93.69 ± 2.27 a	74.37 ± 1.64 b	91.47 ± 3.24 a	94.59 ± 1.74 a
Trifloxystrobin	0.05	6.18 ± 2.78 c	19.68 ± 2.03 b	13.24 ± 2.74 bc	11.39 ± 2.22 bc	18.92 ± 2.84 b
	0.5	28.37 ± 1.79 b	38.77 ± 3.05 a	27.30 ± 2.72 b	14.56 ± 2.37 c	22.30 ± 1.92 bc
	1.0	41.14 ± 3.39 a	48.82 ± 2.41 a	31.32 ± 2.73 b	28.48 ± 2.90 b	34.46 ± 1.76 b
	5.0	56.56 ± 2.54 a	50.43 ± 2.64 a	38.17 ± 3.39 b	43.99 ± 1.62 b	36.49 ± 2.13 b
	10.0	62.79 ± 2.33 a	53.39 ± 1.54 a	42.26 ± 2.86 b	50.32 ± 2.83 a	54.73 ± 2.23 a
Thiophanate-methyl	0.05	8.61 ± 2.64 c	9.92 ± 1.82 c	6.01 ± 1.82 c	16.46 ± 2.24 b	8.11 ± 3.45 c
	0.5	11.77 ± 2.38 bc	17.49 ± 2.81 b	9.28 ± 1.72 c	24.68 ± 3.14 a	18.92 ± 2.44 b
	1.0	24.93 ± 3.48 a	18.86 ± 2.01 b	14.77 ± 2.81 bc	27.22 ± 1.69 a	22.30 ± 3.45 b
	5.0	35.61 ± 1.70 a	21.61 ± 2.43 b	26.90 ± 1.78 ab	34.81 ± 3.18 a	28.38 ± 2.71 ab
	10.0	54.30 ± 1.92 a	25.74 ± 1.99 c	35.13 ± 1.89 b	38.61 ± 1.69 b	32.43 ± 2.98 b

Mean mycelial growth inhibition (%) ± standard deviation (*n* = 4) for five *V. inaequalis* isolates at five concentrations of each fungicide after 28 days of incubation. Different letters within rows indicate significant differences between isolates at the same concentration according to Duncan’s multiple range test (*p* ≤ 0.05).

**Table 3:** EC_50_ values (µg/mL ± SD), statistical groupings, and resistance status of five *V. inaequalis* isolates to three fungicides.

Isolate	Difenoconazole EC_50_ ± SD (Group)	Status	Trifloxystrobin EC_50_ ± SD (Group)	Status	Thiophanate-Methyl EC_50_ ± SD (Group)	Status
ViIF01	0.43 ± 0.04 (bc)	R	2.94 ± 0.29 (a)	R	14.84 ± 1.48 (a)	MR
ViAZ	0.51 ± 0.05 (c)	R	3.60 ± 0.36 (b)	R	1237.20 ± 12.37 (e)	HR
ViIM	1.46 ± 0.15 (d)	R	29.62 ± 2.96 (e)	R	132.22 ± 13.22 (c)	HR
ViHA	0.05 ± 0.01 (a)	S	12.56 ± 1.26 (c)	R	126.66 ± 12.67 (b)	HR
ViEN01	0.36 ± 0.04 (b)	R	15.17 ± 1.52 (d)	R	193.51 ± 19.35 (d)	HR

EC_50_ values are expressed as means ± standard deviation (*n* = 4). Different letters within the same fungicide column indicate significant differences among isolates according to Duncan’s multiple range test (*p* ≤ 0.05). Resistance status: S = sensitive, R = resistant, MR = moderately resistant (10 < EC_50_ ≤ 100 µg/mL), HR = highly resistant (EC_50_ > 100 µg/mL). Resistance thresholds: difenoconazole > 0.1 µg/mL; trifloxystrobin > 2 µg/mL; thiophanate-methyl: MR (10–100 µg/mL), HR (> 100 µg/mL) [37]. Statistical differences were assessed using ANOVA followed by Duncan’s test.

**Table 4:** Disease severity (%) and fungicide efficacy (%) of five *V. inaequalis* isolates on detached apple leaves under preventive and curative treatments after 4 weeks of incubation.

Isolate	Fungicide	Concentration (µg/mL)	Preventive Severity (%) (Mean ± SD, Group)	Preventive Efficacy (%) (Mean ± SD, Group)	Curative Severity (%) (Mean ± SD, Group)	Curative Efficacy (%) (Mean ± SD, Group)
ViEN01	Difenoconazole	0.00 (Control)	99.75 ± 0.5 (d)	0.0 ± 0.0 (d)	98.13 ± 0.5 (d)	0.0 ± 0.0 (d)
		0.05	57.40 ± 2.1 (c)	41.5 ± 2.1 (c)	81.76 ± 3.0 (c)	16.7 ± 3.1 (c)
		0.5	48.98 ± 1.8 (bc)	50.1 ± 1.8 (bc)	74.54 ± 2.5 (bc)	24.0 ± 2.5 (bc)
		1	32.56 ± 1.5 (ab)	66.8 ± 1.5 (ab)	64.18 ± 2.8 (ab)	34.6 ± 2.9 (ab)
		5	21.67 ± 1.2 (a)	77.9 ± 1.2 (a)	43.49 ± 1.9 (a)	55.7 ± 1.9 (a)
		10	14.20 ± 0.9 (a)	85.5 ± 0.9 (a)	27.16 ± 1.4 (a)	72.3 ± 1.4 (a)
	Trifloxystrobin	0.00 (Control)	98.13 ± SD (d)	0.0 ± 0.0 (d)	98.13 ± SD (d)	0.0 ± 0.0 (d)
		0.05	86.64 ± SD (c)	11.7 ± SD (c)	90.86 ± SD (c)	7.4 ± SD (c)
		0.5	77.87 ± SD (bc)	20.6 ± SD (bc)	83.56 ± SD (bc)	14.8 ± SD (bc)
		1	76.09 ± SD (bc)	22.5 ± SD (bc)	78.21 ± SD (bc)	20.3 ± SD (bc)
		5	66.57 ± SD (b)	32.1 ± SD (b)	75.42 ± SD (b)	23.1 ± SD (b)
		10	48.90 ± SD (a)	50.2 ± SD (a)	59.55 ± SD (a)	39.3 ± SD (a)
	Thiophanate-methyl	0.00 (Control)	98.13 ± 2.0 (d)	0.0 ± 2.0 (d)	98.13 ± 2.0 (d)	0.0 ± 2.0 (d)
		0.05	85.45 ± 2.0 (c)	12.9 ± 2.0 (c)	87.24 ± 2.0 (c)	11.1 ± 2.0 (c)
		0.5	80.60 ± 2.0 (bc)	17.9 ± 2.0 (bc)	84.11 ± 2.0 (bc)	14.3 ± 2.0 (bc)
		1	74.61 ± 2.0 (b)	23.9 ± 2.0 (b)	80.91 ± 2.0 (b)	17.5 ± 2.0 (b)
		5	55.54 ± 2.0 (a)	43.4 ± 2.0 (a)	79.95 ± 2.0 (a)	18.5 ± 2.0 (a)
		10	57.41 ± 2.0 (a)	41.5 ± 2.0 (a)	75.78 ± 2.0 (a)	22.7 ± 2.0 (a)
ViIF01	Difenoconazole	0.00 (Control)	98.81 ± 0.5 (d)	0.0 ± 0.0 (d)	98.81 ± 0.5 (d)	0.0 ± 0.0 (d)
		0.05	85.19 ± 2.1 (c)	13.8 ± 2.1 (c)	90.26 ± 3.0 (c)	8.6 ± 3.1 (c)
		0.5	71.30 ± 1.8 (bc)	27.8 ± 1.8 (bc)	83.15 ± 2.5 (bc)	15.8 ± 2.5 (bc)
		1	61.08 ± 1.5 (ab)	38.2 ± 1.5 (ab)	70.72 ± 2.8 (ab)	28.4 ± 2.9 (ab)
		5	59.10 ± 1.2 (a)	40.2 ± 1.2 (a)	63.01 ± 1.9 (a)	36.2 ± 1.9 (a)
		10	46.88 ± 0.9 (a)	52.5 ± 0.9 (a)	51.42 ± 1.4 (a)	47.9 ± 1.4 (a)
	Trifloxystrobin	0.00 (Control)	98.81 ± 0.5 (d)	0.0 ± 0.0 (d)	98.81 ± 0.5 (d)	0.0 ± 0.0 (d)
		0.05	87.67 ± 2.1 (c)	11.3 ± 2.1 (c)	96.21 ± 3.0 (c)	2.6 ± 3.1 (c)
		0.5	80.88 ± 1.8 (bc)	18.1 ± 1.8 (bc)	88.69 ± 2.5 (bc)	10.2 ± 2.5 (bc)
		1	71.96 ± 1.5 (ab)	27.2 ± 1.5 (ab)	74.58 ± 2.8 (ab)	24.5 ± 2.9 (ab)
		5	65.87 ± 1.2 (a)	33.3 ± 1.2 (a)	69.00 ± 1.9 (a)	30.2 ± 1.9 (a)
		10	44.94 ± 0.9 (a)	54.5 ± 0.9 (a)	56.72 ± 1.4 (a)	42.6 ± 1.4 (a)
	Thiophanate-methyl	0.00 (Control)	98.81 ± 0.5 (d)	0.0 ± 0.0 (d)	98.81 ± 0.5 (d)	0.0 ± 0.0 (d)
		0.05	88.86 ± 2.1 (c)	10.1 ± 2.1 (c)	97.75 ± 3.0 (c)	1.1 ± 3.1 (c)
		0.5	84.47 ± 1.8 (bc)	14.5 ± 1.8 (bc)	89.90 ± 2.5 (bc)	9.0 ± 2.5 (bc)
		1	76.06 ± 1.5 (ab)	22.9 ± 1.5 (ab)	80.54 ± 2.8 (ab)	18.5 ± 2.9 (ab)
		5	61.70 ± 1.2 (a)	37.6 ± 1.2 (a)	71.36 ± 1.9 (a)	27.7 ± 1.9 (a)
		10	55.44 ± 0.9 (a)	43.9 ± 0.9 (a)	62.70 ± 1.4 (a)	36.5 ± 1.4 (a)
ViAZ	Difenoconazole	0.00 (Control)	99.75 ± 0.5 (d)	0.0 ± 0.0 (d)	99.75 ± 0.5 (d)	0.0 ± 0.0 (d)
		0.05	87.87 ± 1.8 (c)	11.9 ± 1.8 (c)	88.67 ± 2.0 (c)	11.1 ± 2.0 (c)
		0.5	66.10 ± 1.5 (bc)	33.7 ± 1.5 (bc)	76.20 ± 1.9 (bc)	23.6 ± 1.9 (bc)
		1	45.89 ± 1.2 (ab)	54.0 ± 1.2 (ab)	54.09 ± 1.5 (ab)	45.7 ± 1.5 (ab)
		5	40.01 ± 0.9 (a)	59.9 ± 0.9 (a)	40.31 ± 1.0 (a)	59.6 ± 1.0 (a)
		10	27.97 ± 0.7 (a)	71.9 ± 0.7 (a)	39.04 ± 0.8 (a)	60.9 ± 0.8 (a)
	Trifloxystrobin	0.00 (Control)	99.75 ± 0.5 (d)	0.0 ± 0.0 (d)	99.75 ± 0.5 (d)	0.0 ± 0.0 (d)
		0.05	94.86 ± 2.0 (c)	4.9 ± 2.0 (c)	92.58 ± 2.2 (c)	7.2 ± 2.2 (c)
		0.5	86.76 ± 1.8 (bc)	13.0 ± 1.8 (bc)	83.86 ± 1.9 (bc)	15.9 ± 1.9 (bc)
		1	78.03 ± 1.5 (ab)	21.7 ± 1.5 (ab)	69.07 ± 1.6 (ab)	30.7 ± 1.6 (ab)
		5	75.00 ± 1.2 (a)	24.8 ± 1.2 (a)	55.93 ± 1.3 (a)	43.9 ± 1.3 (a)
		10	68.76 ± 0.9 (a)	31.1 ± 0.9 (a)	50.18 ± 1.0 (a)	49.7 ± 1.0 (a)
	Thiophanate-methyl	0.00 (Control)	99.75 ± 0.5 (d)	0.0 ± 0.0 (d)	99.75 ± 0.5 (d)	0.0 ± 0.0 (d)
		0.05	90.00 ± 2.0 (c)	9.8 ± 2.0 (c)	95.49 ± 2.2 (c)	4.3 ± 2.2 (c)
		0.5	89.84 ± 1.8 (bc)	10.0 ± 1.8 (bc)	92.83 ± 1.9 (bc)	6.9 ± 1.9 (bc)
		1	85.87 ± 1.5 (ab)	13.9 ± 1.5 (ab)	87.22 ± 1.6 (ab)	12.5 ± 1.6 (ab)
		5	79.67 ± 1.2 (a)	20.1 ± 1.2 (a)	80.09 ± 1.3 (a)	19.7 ± 1.3 (a)
		10	77.33 ± 0.9 (a)	22.5 ± 0.9 (a)	79.86 ± 1.0 (a)	19.9 ± 1.0 (a)
ViIM	Difenoconazole	0.00 (Control)	98.88 ± 0.5 (d)	0.0 ± 0.0 (d)	98.88 ± 0.5 (d)	0.0 ± 0.0 (d)
		0.05	85.07 ± 2.0 (c)	13.9 ± 2.0 (c)	90.00 ± 3.0 (c)	8.9 ± 3.1 (c)
		0.5	79.10 ± 1.8 (bc)	20.0 ± 1.8 (bc)	82.73 ± 2.5 (bc)	16.3 ± 2.5 (bc)
		1	67.08 ± 1.5 (ab)	32.1 ± 1.5 (ab)	74.54 ± 2.8 (ab)	24.6 ± 2.9 (ab)
		5	52.13 ± 1.2 (a)	47.3 ± 1.2 (a)	61.08 ± 1.9 (a)	38.2 ± 1.9 (a)
		10	38.87 ± 0.9 (a)	60.7 ± 0.9 (a)	48.66 ± 1.4 (a)	50.8 ± 1.4 (a)
	Trifloxystrobin	0.00 (Control)	98.88 ± 4.94 (d)	0.0 ± 0.0 (d)	98.88 ± 4.94 (d)	0.0 ± 0.0 (d)
		0.05	90.00 ± 4.50 (c)	9.0 ± 4.5 (c)	84.77 ± 4.24 (c)	14.2 ± 4.2 (c)
		0.5	85.89 ± 4.29 (bc)	13.1 ± 4.3 (bc)	75.00 ± 3.75 (bc)	24.1 ± 3.8 (bc)
		1	79.65 ± 3.98 (ab)	19.5 ± 4.0 (ab)	70.01 ± 3.50 (ab)	29.2 ± 3.5 (ab)
		5	73.43 ± 3.67 (a)	25.7 ± 3.7 (a)	65.40 ± 3.27 (a)	33.8 ± 3.3 (a)
		10	69.90 ± 3.50 (a)	29.3 ± 3.5 (a)	52.65 ± 2.63 (a)	46.7 ± 2.6 (a)
	Thiophanate-methyl	0.00 (Control)	98.88 ± 4.94 (d)	0.0 ± 0.0 (d)	98.88 ± 4.94 (d)	0.0 ± 0.0 (d)
		0.05	97.65 ± 4.88 (c)	1.2 ± 4.9 (c)	98.27 ± 4.91 (c)	0.6 ± 4.9 (c)
		0.5	90.87 ± 4.54 (bc)	8.1 ± 4.5 (bc)	92.79 ± 4.64 (bc)	6.1 ± 4.6 (bc)
		1	83.31 ± 4.17 (ab)	15.7 ± 4.2 (ab)	76.79 ± 3.84 (ab)	22.3 ± 3.8 (ab)
		5	78.17 ± 3.91 (a)	20.9 ± 3.9 (a)	74.25 ± 3.71 (a)	24.9 ± 3.7 (a)
		10	70.90 ± 3.55 (a)	28.3 ± 3.5 (a)	73.65 ± 3.68 (a)	25.5 ± 3.7 (a)
ViHA	Difenoconazole	0.00 (Control)	99.20 ± 0.5 (d)	0.0 ± 0.0 (d)	99.20 ± 0.5 (d)	0.0 ± 0.0 (d)
		0.05	81.00 ± 2.0 (c)	18.3 ± 2.0 (c)	82.32 ± 3.0 (c)	17.0 ± 3.1 (c)
		0.5	68.34 ± 1.8 (bc)	31.1 ± 1.8 (bc)	72.27 ± 2.5 (bc)	27.1 ± 2.5 (bc)
		1	57.47 ± 1.5 (ab)	42.0 ± 1.5 (ab)	70.32 ± 2.8 (ab)	29.1 ± 2.9 (ab)
		5	50.25 ± 1.2 (a)	49.3 ± 1.2 (a)	50.24 ± 1.9 (a)	49.4 ± 1.9 (a)
		10	30.90 ± 0.9 (a)	68.8 ± 0.9 (a)	40.87 ± 1.4 (a)	58.8 ± 1.4 (a)
	Trifloxystrobin	0.00 (Control)	99.20 ± 4.96 (d)	0.0 ± 0.0 (d)	99.20 ± 4.96 (d)	0.0 ± 0.0 (d)
		0.05	85.09 ± 4.25 (c)	14.2 ± 4.3 (c)	98.87 ± 4.94 (c)	0.3 ± 4.9 (c)
		0.5	80.65 ± 4.03 (bc)	18.7 ± 4.0 (bc)	91.67 ± 4.58 (bc)	7.6 ± 4.6 (bc)
		1	68.87 ± 3.44 (ab)	30.6 ± 3.4 (ab)	87.98 ± 4.40 (ab)	11.2 ± 4.4 (ab)
		5	62.60 ± 3.13 (a)	36.9 ± 3.1 (a)	79.89 ± 3.99 (a)	19.5 ± 4.0 (a)
		10	50.16 ± 2.51 (a)	49.4 ± 2.5 (a)	68.50 ± 3.43 (a)	30.9 ± 3.4 (a)
	Thiophanate-methyl	0.00 (Control)	99.20 ± 4.96 (d)	0.0 ± 0.0 (d)	99.20 ± 4.96 (d)	0.0 ± 0.0 (d)
		0.05	93.33 ± 4.67 (c)	5.9 ± 4.7 (c)	87.73 ± 4.39 (c)	11.6 ± 4.4 (c)
		0.5	91.70 ± 4.59 (bc)	7.6 ± 4.6 (bc)	76.68 ± 3.83 (bc)	22.7 ± 3.8 (bc)
		1	80.22 ± 4.01 (ab)	19.1 ± 4.0 (ab)	68.44 ± 3.42 (ab)	30.9 ± 3.4 (ab)
		5	78.31 ± 3.92 (a)	21.0 ± 3.9 (a)	66.68 ± 3.33 (a)	32.8 ± 3.3 (a)
		10	73.50 ± 3.68 (a)	25.9 ± 3.7 (a)	50.16 ± 2.51 (a)	49.4 ± 2.5 (a)

Disease severity (%) was assessed 4 weeks after inoculation using the scale of Calenge et al. [35] Efficacy (%) was calculated as: [(control severity−treatment severity)/control severity] × 100. Values are means ± standard deviation (*n* = 4). Different letters within columns indicate significant differences according to Duncan’s multiple range test (*p* ≤ 0.05). Preventive: leaves treated before inoculation; curative: leaves treated after inoculation. All concentrations are expressed in µg/mL.

## Data Availability

The original contributions presented in this study are included in the article. Further inquiries can be directed to the corresponding author.
